# Estimation of the value of curative therapies in oncology: a willingness-to-pay study in China

**DOI:** 10.1186/s12962-023-00442-y

**Published:** 2023-06-05

**Authors:** Li Huang, Xiaochen Peng, Lihua Sun, Dawei Zhang

**Affiliations:** 1 School of Business Administration, Shenyang Pharmaceutical University, No. 103, Wenhua Rd, Shenhe Dist, Shenyang, 110016 Liaoning People’s Republic of China; 2 Shanghai Health Development Research Center, No. 602, Jianguo Rd, Jingan Dist, Shanghai, 200031 People’s Republic of China

## Abstract

The objective of this study was to estimate the willingness to pay (WTP) per quality-adjusted life year (QALY) among people with malignancies in China. The WTP for a QALY was estimated using a contingent valuation survey. Health utility was measured in EuroQol-5 dimensions (EQ-5D). The questionnaires were completed in face-to-face interviews. Respondents consisted of patients with malignant tumors and their family members and came from three tertiary hospitals in different cities with high, medium, and low gross domestic product (GDP) levels. In this study, we offered lump-sum payments and 10 year installment plans to respondents. Finally, we conducted sensitivity analysis and stepwise regression analyses to identify factors that affected the WTP/QALY ratios. A total of 1264 people participated in this survey, and 1013 people gave WTP responses for further analysis. The mean and median WTP/QALY values based on the lump-sum payments were 366,879 RMB (53,171USD, 5.1 times the GDP per capita) and 99,906 RMB (14,479USD, 1.39 times the GDP per capita) for the overall sample; 339,330 RMB (49,178USD, 4.71 times the GDP per capita) and 83,875 RMB (12,156USD, 1.16 times the GDP per capita) for the patient group; and 407,396 RMB (59,043USD, 5.66 times the GDP per capita) and 149,436 RMB (21,657USD, 2.08 times the GDP per capita) for the family group. Considering the skewedness of the data distribution, we suggest setting the cost-utility threshold with reference to the median. When the payment plan changed to 10-year installments, the median increased to 134,734RMB (19,527USD), 112,390 RMB (16,288USD) and 173,838 RMB (25,194USD) for the above groups, respectively. EQ-5D-5L health utility, annual household income per capita, patients with other chronic diseases, occupation, regular physical examinations (patients) and age (family members) were significantly related to WTP/QALY. This study provides empirical evidence of the monetary value of a QALY from a sample of the Chinese population with malignancies. In addition, the ratio of the WTP/QALY to GDP per capita was related to the disease and hypothetical scenario, and a higher ratio of GDP per capita for malignant tumor therapies should be considered.

**Supplementary Information:**

The online version contains supplementary material available at 10.1186/s12962-023-00442-y.

## Introduction

According to the global cancer burden study [1], there would be approximately 19.3 million new cases and 10 million deaths worldwide in 2020, with Asia having a much higher incidence and death rate than other continents, accounting for approximately 49.3% and 58.3% of the world, respectively (Europe accounted for about 22.8% and 19.6% of the global total, and the Americas for about 20.9% and 14.2%). China accounted for approximately 23.7% and 30.2% of new cases and deaths worldwide, respectively [2], and China has the highest number of new malignant tumor cases and deaths in the world. According to the National Cancer Center's analysis [3], the incidence rate of malignant tumors had increased by 3.9% per year over the last ten years, while the mortality rate has increased by 2.5% per year. It is also predicted that [4], the death toll of malignant tumors in China will rise from 2,433,700 cases in 2015 to 3,618,700 cases in 2030. With the increase of incidence rate and mortality, malignant tumors have become the primary cause of death and the main public health problem in China [5].

While, the treatment of malignant tumors has imposed a significant financial burden on patients' families as well as national medical insurance. In a nationwide study conducted by the National Cancer Center, the average medical cost per patient was about 9,739USD [6], exceeding the GDP per capita of the study year. According to the National Cancer Center's publication "Cancer Statistics in China, 2015," as cancer incidence rates climbed, more than 220 billion RMB would be spent on relevant medical treatment each year [3].

In recent years, the China Health Security Administration (NHSA) has implemented a number of initiatives to improve accessibility and affordability of medication for patients, including national reimbursement drug list negotiation (NRDL), which are well known among patients and their families due to its direct impact on the drug prices. Anti-tumor drugs’ price negotiations have attracted a lot of attention. Each year between 2018 and 2022, nearly 20 anti-tumor drugs were added to the NRDL (17 in 2018, 22 in 2019, 17 in 2020, 18 in 2021, and 23 in 2022) [7], including the drugs that were expensive but innovative with clear efficacy, such as Abiraterone and Toripalimab. In particular, on June 23, 2021, China NMPA (national medical products administration) approved the marketing of the first CAR T-cell therapy drug Axicabtagene ciloleucel (trade name: Yescarta) [8]. Many have been greatly shocked by both the treatment's cancer-free success rates and its expensive price (1.2 million yuan each bag) [9]. During the past two years, this type of therapy had passed the preliminary review process, but it all halted at the comprehensive review process and did not enter the final negotiation process.

In NRDL negotiation, manufacturers are required to provide pharmacoeconomic evaluation (mostly are cost utility analysis,CUA and cost effectiveness analysis, CEA) to provide evidence for experts in price review process to conduct discount price calculation and estimation, but there is no official ICER threshold in NRDL negotiation yet.

In recent years, there has been some research on the threshold in the context of China. He Wei [10] and Ochalek J [11] used the opportunity cost approach to estimate the opportunity cost of health resources from the perspective of supply, which is 38,100–45,500 RMB/DALY and 27,923–52,247 RMB/DALY, respectively; From the perspective of demand, Ye Ziping et al. [12] used the contingent valuation method (CVM) to estimate the WTP for a QALY, which was 113,120 RMB, while Cai et al. [13] calculated the statistical life value of a QALY to be 1.45 times the GDP per capita. However, it is uncertain if the threshold for treating malignant tumors should be the same as it is for other diseases, particularly for breakthrough treatments such as CAR T-cell therapy. In China, there is no threshold research based on the perspective of cancer patients. Thus, we employ the same CVM approach as conducted in previous studies, so as to enable the comparison of study results, to explore the willingness to pay per QALY for the hypothetical curative therapy from the perspective of cancer patients and their family members.

## Methods

### Study design

According to the interpretation of the “Global Cancer Report, 2020” by Zongchao Liu et al. [2] and the China Cancer Registry Annual Report 2019 [14], the most commonly diagnosed cancers in China were lung cancer (17.9% of total cases), colorectal cancer (12.2%), gastric cancer (10.5%), breast cancer (9.1%), and liver cancer (9%); 54.2% of patients were male and 45.8% were female; 54% were urban patients and 46% were rural patients; the incidence of malignant tumors was highest in the east region (53.4%), compared with the central and western regions (23.9% and 22.7%). Taking into account the geographical divisions of China, the distribution of GDP per capita in 2019 (32,995–164,220 RMB (4775–23,766USD), GDP per capita: 70,892 RMB (10,259USD),^1^) [15], and the resources available, we selected three cities: Ningbo, Zhejiang (East China, GDP per capita: 143,157 RMB (20,717USD)) [16], Shenyang, Liaoning (Northeast China, GDP per capita: 77,777 RMB (11,256USD)) [17], and Huaihua, Hunan (South Central China, connected to the western regions, GDP per capita: 32,453 RMB (4,697USD)) [18].

Before performing a formal inquiry, we asked various cancer physicians to assess the questionnaire's content for potential harmful impact on or other sensitive information about the patient, and we amended the questionnaire based on their opinion. Following that, a pilot study was carried out among 32 patients and their families from the provinces of Zhejiang, Liaoning, and Hunan in terms of WeChat or face-to-face method to determine the WTP distribution and to examine the feasibility of the study.

Lastly, we selected a tertiary hospital in each of the above cities and performed the face-to-face interview on respondents who met the criteria and were willing to participate in the survey from November 2020 to February 2022. The survey was carried out by graduate students majoring in pharmacy administration (pharmacoeconomics) at Shenyang Pharmaceutical University, who had received detailed training in conducting interviews. The interview would begin after informed consent, and respondents could exit the survey at any time. The questionnaires were ethically approved (KY-2020110601, 2021-R104, 20220104).

### Recruitment criteria and sample size

Recruitment criteria: For the respondents participating in the survey, the following requirements were need to be met: (1) patients who had been diagnosed with malignant tumor diseases; (2) participants who understood that their or their family member's disease was malignant cancer; (3) Chinese citizens; (4) aged between 18 and 75; (5) patients who showed clear awareness, was able to communicate smoothly with others, and was able to listen, speak and write; (6) participants who were volunteered to participate in the interview.

The minimum sample size was 384 using Formula (1) and considering nonresponses, it was increased to 400 for both the patient and family groups. Formula (1) is as follows [19]:

1 $${\text{n}} = Z^{2} (1\_{\alpha \mathord{\left/ {\vphantom {\alpha {2)p{{{\text{(1\_P)}}} \mathord{\left/ {\vphantom {{{\text{(1\_P)}}} {\text{d}}}} \right. \kern-0pt} {\text{d}}}}}} \right. \kern-0pt} {2)p{{{\text{(1\_P)}}} \mathord{\left/ {\vphantom {{{\text{(1\_P)}}} {\text{d}}}} \right. \kern-0pt} {\text{d}}}}}^{2}$$

w*here “n” represents the required minimum sample size; “p” is the expected proportion of a certain feature in the malignant tumor population, which is generally set to 50% when the data are unavailable; “d” represents the absolute precision, which was assigned a value of 0.05; and “Z*_*(1-α/2)*_*” equates to 1.96 (α*=*5%).*

### Questionnaire design

We had conducted several cost-utility threshold related studies in the patients with malignant tumor in China, the research topics contained maintenance therapy and curative treatment. This study was one of our studies on the cost-utility threshold for patients with malignant tumors in China, which mainly involved three fields: health utility measurement, WTP estimation, and subgroup analysis based on demographic characteristics.

#### Health status utility measurements

The EuroQol-5 dimensions (EQ-5D-5L) were used to measure the health utility of patients with malignant tumors. The EQ-5D-5L is a general multi-attribute health status measurement tool that includes five dimensions: mobility, self-care, usual activity, pain or discomfort, and anxiety or depression. Each dimension is described in five levels: no problems, slight problems, moderate problems, severe problems, and unable to/extreme problems. A score of “11111” indicates full health status, and a score of “55555” indicates the worst health status. The health utility scores in our study were calculated using a Chinese population-based value set from published studies [20].

#### WTP measurement

A payment card combined with open-ended questions was used in the questionnaire. The range of offered bids on the payment card for curative treatment payments was determined as RMB 0, RMB 300,000, RMB 600,000, RMB 900,000, RMB 1,200,000, RMB 1,500,000 and RMB 1,800,000 based on our pilot survey and the costs of treatment for malignant tumors reported by studies published in the CNKI during 2010–2017.

The assumption under a hypothetical treatment scenario was stated to each respondent: The average life expectancy of Chinese people is 80 years. Assuming the disease you (or your family) are currently hospitalized for is a chronic condition, the disease can be fully cured by taking a new, painless, nontoxic and side effect free therapy without additional treatments (such as surgery, chemotherapy or radiation, etc.). If you do not accept this novel therapy, you current state of health will be maintained. Are you willing to pay for this novel therapy at your own expense? If the respondent was willing to pay for the novel therapy, the range of the bidding amount was determined through a payment card, and then the maximum amount was determined through open-ended questions. If the respondent was not willing to pay for the novel therapy, the reason was further addressed. To increase the reliability of responses, the respondents were reminded beforehand of their financial ability and asked afterward about their source of income [21].

In addition to lump-sum payments, we offered a 10 year installment plan to respondents below 70 years of age, and the range of bids for yearly payments was set as RMB 0, RMB 30,000, RMB 60,000, RMB 90,000, RMB 120,000, RMB 150,000 and RMB 180,000, referencing the survey on WTP among patients with malignant tumors in our other study (unpublished). A sample CV task was provided as Additional file 1.

#### Demographic characteristics

In addition to sex, age, place of residence, marital status, education level, annual household income and health insurance plan, characteristics that could have an impact on willingness to pay, such as regular physical examinations, co-occurring chronic diseases, etc., were included in our survey and showed in Table 1.

**Table 1:** Patient characteristics

Demographics		Value
Sex, N (%)	Female	544 (53.7%)
	Male	469 (46.3%)
Age	Mean (SD)	56.2 (11.6)
Residence	Rural	450 (44.4%)
	Urban	563 (55.6%)
Malignant tumor type	Colorectal cancer	160 (15.8%)
	Lung cancer	142 (14%)
	Breast cancer	142 (14%)
	Gastric carcinoma	92 (9.1%)
	Cervical cancer	89 (8.8%)
	Nasopharyngeal carcinoma	57 (5.6%)
	Lymphoma	47 (4.6%)
	Leukemia	40 (3.9%)
	Prostate cancer	22 (2.2%)
	Esophageal cancer	31 (3.1%)
	Thyroid cancer	33 (3.3%)
	Myeloma	22 (2.2%)
	liver cancer	18 (1.8%)
	Pancreatic cancer	22 (2.2%)
	Brain cancer	13 (1.3%)
	Other	83 (8.2%)
Education level	Elementary school and below	308 (30.4%)
	Junior high school	356 (35.1%)
	High School and secondary School	218 (21.5%)
	College and above	131 (12.9%)
Health insurance	National medical insurance for urban employees	412 (40.7%)
	National medical insurance for urban and rural residents	530 (52.3%)
	Commercial medical insurance	66 (6.5%)
	No insurance	5 (0.5%)
Marital status	Unmarried	29 (2.9%)
	Married	893 (88.2%)
	Divorced	16 (1.6%)
	Widowed	75 (7.4%)
Working status	Employed full-time	138 (13.6%)
	Employed part-time	50 (4.9%)
	Self-employed	83 (8.2%)
	Housewife	143 (14.1%)
	Farming	176 (17.4%)
	Student	5 (0.5%)
	Unemployed	120 (11.8%)
	Retired	296 (29.2%)
	Other	2 (0.2%)
Annual household income per capita (RMB)	12,000 and below	182 (18%)
	12,001–20,000	177 (17.5%)
	20,001–35,000	253 (25%)
	35,001–60,000	240 (23.7%)
	60,001 and above	161 (15.9%)
Regular physical examinations	Y	380 (37.5%)
	N	633 (62.5%)
Other chronic diseases	Y	281 (27.7%)
	N	732 (72.3%)
Hospitalization due to other diseases in the past year	Y	54 (5.3%)
	N	959 (94.7%)
Family member was severely ill/passed away in the past year	Y	55 (5.4%)
	N	958 (94.6%)
Family member has/had similar cancers	Y	260 (25.7%)
	N	753 (74.3%)
Family member has other diseases	Y	207 (20.4%)
	N	806 (79.6%)
EQ-5D-5L	Mean (SD)	0.749 (0.199)
	Median	0.806

### Data analysis

Equation 2 was employed in calculating the WTP for a QALY.

2 $$WTP/QALY=WTP/{\sum }_{t=1}^{remain \,life \,years}(cured \,population\, health\, utility\, value - current\, health\, utility)*{(1+r)}^{t}$$

In the equation, WTP was included in the lump-sum payment; the denominator indicated the health benefit of the curative treatment; for patients with other chronic diseases (hypertension, diabetes, etc.), the health utility value for the cure adapted the utility data from the study among patients with chronic diseases in Suzhou that used EQ-5D-3L measurement [22], and for patients without other chronic diseases, the health utility after curative treatment was 1. The discount rate was 5% based on the China pharmacoeconomic evaluation guidelines [23]. The WTP/QALY was represented by WTP/QALY _(r=5%)_. The value of WTP/QALY was calculated using Eq. 2.

Stepwise regression analysis was conducted to examine the effect of patient demographic characteristics on the WTP/QALY _(r=5%)_ value by using a generalized linear model with gamma distribution and log function. The statistical analysis was carried out using IBM SPSS 23 and Stata 17.

## Results

### Descriptive analysis

A total of 1264 respondents participated in the survey, 56 refused to answer, 37 were willing to pay money for the hypothetical curative therapy but failed to give an exact amount, and 143 were not willing to pay due to affordability, suggesting that the government should bear the entire expense and others, 15 respondents had health utility scores were equal to 1, Finally, 1013 questionnaires were valid and analyzed in our study. A total of 59.5% of the overall sample were patients themselves, and 40.5% were their family members.

In our study, the average age of the patients was 56.2 years, slightly younger than the average age of cancer incidence in China (61.3 years) [24]; female respondents accounted for 53.7%, which was different from the gender ratio in cancer patients in China (54.2% were males and 45.8% were females). 44.4% of the patients lived in in rural areas, and 55.6% were urban residents, which was close to the ratios of cancer patients in China. Among the 5 most common cancers in China, lung cancer (14%), colorectal cancer (15.8%), gastric cancer (9.1%), and breast cancer (14%) accounted for approximately 52.9% of the overall cancer cases in our study, but the cases of liver cancer (18 cases) were small in amount in our study. In addition, the distribution of annual per capita household income roughly approximated the income distribution of the Chinese population [25], while annual household income per capita in the range of 20,001–35,000 RMB and 35,001–60,000 RMB accounted for 25% and 23.7%, respectively, slightly higher than others.The mean and median health utility values of the patients were 0.749 and 0.806 as measured by the EQ-5D-5L, respectively, it was similar to the results measured (EQ-5D-3L) by Yan Xiaoling et al. [26] in patients in Beijing, the mean and median utility values were 0.759 and 0.795. Other characteristics including occupation, regular medical checkups, and whether they had other diseases were also described in this study. The demographic characteristics of the patients are shown in Table 1.

### Willingness to pay

80.1% of total respondents (N = 1264) gave positive WTP values, and we analyzed the WTP of the sample. The mean and median WTP values were 343,897RMB (49,840USD) and 200,000 RMB (28,986USD),^2^ respectively. In the subgroup analysis, the mean and median WTP values for the patient group were 304,929 RMB (44,193USD) and 200,000 RMB (28,986USD), respectively, which were lower than those for the family member group, namely, 401,207 RMB (58,146USD) and 300,000 RMB (43,478USD). According to the M-W(U) test, the difference between the two groups was statistically significant (Z = − 5.834, P < 0.05).

### WTP/QALY_(r=5%)_

The WTP/QALY value and ratio of WTP/QALY_(r=5%)_ to China’s GDP per capita in 2020 (72,000 RMB) [25] are shown in Table 2. The mean WTP/QALY_(r=5%)_ values were higher than the median values in the overall sample, patient group and family member group, indicating a skewed distribution. In the comparison of WTP/QALY_(r=5%)_ values for the patient group with those for the family member group by the M-W(U) test, a significant difference was found between the two groups (Z = − 5.420, P < 0.05).

**Table 2:** The mean (GDP ratio) and median (GDP ratio) of WTP/QALY_(r=5%)_ of surveyed samples

Statistic	Overall sample	Patient group		Family member group
Mean (GDP ratio)	366,879 (5.1)	339,330 (4.71)		407,396 (5.66)
Median (GDP ratio)	99,906 (1.39)	83,875 (1.16)		149,436 (2.08)
Minimum	97	97	282	
Maximum	29,906,174	29,906,174	11,787,367	
25th percentile	39,130	32,990	56,482	
75th percentile	275,715	225,888	377,004	
Z	/	− 5.420		
P	/	0.000		

Afterwards, sensitivity analysis was carried out. First, after eliminating the outliers (bottom 5% and top 5% of the range of health benefit and WTP values), we found that when the extreme values of both the health utility gain and WTP were excluded, the change in the mean value of WTP/QALY was greatest for the overall population, the patient group, and the family member group; the change from excluding the extreme values of health utility gain was greater than it was for WTP, indicating a greater effect on the mean value of WTP/QALY. Sensitivity analysis revealed that the median value of WTP/QALY was not significantly affected by excluding extreme values. In addition,the mean was much higher than the median, indicating a skewed distribution of the WTP/QALY.

Then, according to the Guidelines for Pharmacoeconomic Evaluations in China [23], discount rates of 0%, 3% and 8% were used in the sensitivity analysis. The WTP/QALY values increased as the discount rate increased, implying a positive correlation. The sensitivity analysis results are shown in Tables 3 and 4.

**Table 3:** WTP/QALY _(r=5%)_ sensitivity analysis after eliminating outliers

WTP/QALY	Overall sample			Patient group			Family member group		
	Health benefit	WTP	Health benefit and WTP	Health benefit	WTP	Health benefit and WTP	Health benefit	WTP	Health benefit and WTP
Mean (SD)	226,370 (384,767)	334,891 (1,357,689)	188,731 (255,329)	179,290 (333,366)	298,815 (1,552,180)	147,671 (188,707)	292,960 (415,597)	408,950 (1,008,819)	284,689 (400,241)
Median	99,232	97,634	97,568	81,533	82,770	81,649	149,436	151,315	150,163
Minimum	97	3,350	4539	97	3,350	4,884	3,041	7,998	8,584
Maximum	4,670,063	29,906,170	2,154,274	4,377,320	29,906,170	1,495,627	3,377,792	11,787,370	3,377,792
25th percentile	39,828	40,531	41,385	32,990	35,273	35,273	58,108	58,322	61,193
75th percentile	241,575	239,868	224,524	194,413	201,158	184,305	357,627	369,093	333,289

**Table 4:** WTP/QALY sensitivity analysis at different discount rates

WTP/QALY	Overall sample			Patient group			Family member group		
	0%	3%	8%	0%	3%	8%	0%	3%	8%
Mean (SD)	244,075 (856,537)	313,583 (1,129,520)	455,210 (1,780,175)	210,914(909,834)	283,095 (1,264,650)	433,218 (2,104,909)	292,846 (769,984)	358,423 (894,070)	487,555 (1,148,068)
Median	60,102	83,218	130,974	47,054	68,444	105,476	93,198	127,221	184,029
Minimum	60	81	123	60	81	123	144	221	385
Maximum	12,829,066	22,200,636	43,033,900	12,829,066	22,200,635	43,033,900	10,206,626	11,143,318	12,781,577
25th percentile	23,088	32,670	50,198	18,822	26,858	41,875	34,272	48,322	69,221
75th percentile	172,776	230,277	341,876	122,651	176,298	285,381	262,258	331,604	456,369

### Multiple regression analysis

The results of stepwise regression analysis for patients and family members are shown in Tables 5 and 6. Characteristics including EQ-5D-5L health utility, annual household income per capita and patients with other chronic diseases had a positive relation to WTP/QALY value; however, family members did not have a significantly higher WTP for a QALY when annual household income per capita was 12,001–20,000 RMB. In addition, patients with regular physical examinations were willing to pay more for a QALY gain; and compared to patients with full-time jobs, retired patients had higher WTP/QALY values. Among family members, age was a positively significant factor, and elderly patients had higher WTP/QALY values; compared to patients who were employed full-time, self-employment and students had a significantly higher WTP/QALY ratio, and patients who were employed part-time and farmers had a smaller WTP/QALY ratio, but the difference was not significant (P > 0.1). It worth notice that age was a factor that significantly affect the value of WTP/QALY in family member group but not in the patient group.

**Table 5:** Stepwise regression analysis of WTP/QALY _(r=5%)_ for patients

Variables	WTP/QALY_(r=5%)_			
	Partial regression coefficient	Standard error	Z	P
Constant	− 1.999***	0.356	− 5.61	0.000
EQ-5D-5L	4.119***	0.303	13.61	0.000
Annual household income per capital				
≤ 12,000 RMB	0.000			
12,001–20,000 RMB	0.646***	0.217	2.93	0.003
20,001–35,000 RMB	0.883***	0.203	4.35	0.000
35,001–60,000 RMB	1.245***	0.225	5.54	0.000
≥ 60,001 RMB	2.150***	0.261	8.24	0.000
Other chronic diseases				
N	0.000			
Y	0.966***	0.153	6.31	0.000
Regular physical examinations				
N	0.000			
Y	0.306**	0.144	2.12	0.034
Occupation				
Full-time employment	0.000			
Part-time employment	− 0.227	0.326	− 0.70	0.485
Self-employment	0.450	0.280	1.61	0.108
Housewife	0.318	0.266	1.20	0.232
Farming	0.239	0.265	0.90	0.367
Student	1.142	1.127	1.01	0.311
Unemployment	0.186	0.253	0.74	0.462
Retirement	0.406**	0.206	1.97	0.049
Other	− 2.317	1.566	− 1.48	0.139
AIC	7.475			
Log likelihood	− 2237.849			

^***^P < 0.01

^**^P < 0.05

^*^P < 0.1

**Table 6:** Stepwise regression analysis of WTP/QALY _(r=5%)_ for family members

Variables	WTP/QALY_(r=5%)_			
	Partial regression coefficient	Standard error	Z	P
Constant	− 1.818***	0.486	− 3.74	0.000
EQ-5D-5L	3.252***	0.249	13.08	0.000
Age	0.027***	0.008	3.38	0.001
Annual household income per capita				
≤ 12,000 RMB	0.000			
12,001–20,000 RMB	0.058	0.235	0.25	0.806
20,001–35,000 RMB	0.605***	0.226	2.68	0.007
35,001–60,000 RMB	0.903***	0.224	4.02	0.000
≥ 60,001 RMB	1.322***	0.251	5.27	0.000
Other chronic diseases				
N	0.000			
Y	0.729***	0.154	4.74	0.000
Occupation				
Full-time employment	0.000			
Part-time employment	− 0.538	0.451	− 1.19	0.234
Self-employment	0.633*	0.331	1.92	0.055
Housewife	0.077	0.314	0.24	0.807
Farming	− 0.073	0.311	− 0.24	0.813
Student	2.002**	0.832	2.41	0.016
Unemployment	0.370	0.363	1.02	0.309
Retirement	0.335	0.301	1.11	0.265
Other	− 1.429	1.348	− 1.06	0.289
AIC	8.361			
Log likelihood	− 1698.065			

^***^P < 0.01

^**^P < 0.05

^*^P < 0.1

### 10 year installment payment plan

The 10 year installment payment plan was offered after completing the questionnaire based on lump-sum payments. Patients aged 70 years and above were excluded, which accounted for 13.5% of the overall sample. Approximately 8% of the overall sample refused to answer, 4.34% were unwilling to use the installment payment plan, and approximately 1.3% were willing to pay with the installment plan but failed to give a maximum amount. Therefore, 60.7% of the overall sample provided valid responses to the questions in the 10 year installment payment plan scenario, 375 patients and 240 family members in total.

The discount rate was set as 5%, and a statistically significant difference was found between the lump-sum and 10 year installment payment plans (Z = − 3.627, P < 0.05) by employing the M-W (U) test. There was a statistically significant difference between the patient group and the family member group (Z = − 3.368, P < 0.05). The results for WTP/QALY _(r=5%)_ values in the 10 year installment payment plan scenario are shown in Table 7.

**Table 7:** WTP/QALY _(r=5%)_ values in the 10 year installment payment plan scenario

Payment method	Lump-sum payments		10 year installment payment plan	
Respondents	WTP values for both payment plans		Patients	Family members
Sample size	615	615	375	240
Mean ± SD	321,788 (1405,140)	388,260 (1437,546)	329,374 (1314,842)	480,269 (1,609,267)
Median	98,157	134,734	112,390	173,838
Minimum	805	3,639	3,639	6,176
Maximum	29,906,174	23,092,755	23,092,755	21,381,776
25th percentile	39,359	49,220	44,303	68,883
75th percentile	236,238	317,095	280,605	391,128
Z	− 3.627		− 3.368	
P	0.000		0.001	

## Discussion

In this paper, we estimated the results of the WTP for a QALY from a survey among Chinese individuals with malignancies. The mean and median WTP/QALY values were 366,879 RMB (53,171USD, 5.1 times the GDP per capita) and 99,906 RMB (14,479USD, 1.39 times the GDP per capita) for the overall sample; 339,330 RMB (49,178USD, 4.71 times the GDP per capita) and 83,875 RMB (12,156USD, 1.16 times the GDP per capita) for the patient group; and 407,396 RMB (59,043USD, 5.66 times the GDP per capita) and 149,436 RMB (21,657USD, 2.08 times the GDP per capita) for the family group, respectively. The mean values were much higher than the median values, which showed a skewed distribution of WTP/QALY values, and sensitivity analysis indicated that the median was relatively stable; thus, we suggest setting the cost-utility threshold with reference to the median. The median monetary value of one QALY for the overall group (99,906 RMB, 14,479USD, 1.39 times the GDP per capita) was close to the willingness to pay for a QALY reported from previous studies in China, with 113,120 RMB in the Chinese general population reported in 2020 [12], and the monetary value of a QALY based on the value of statistical life (1.45 times the GDP per capita, 1.16–2.90 times the GDP per capita) [13]. However, these values were smaller than the median in our other study (incurable and maintenance scenario: 177,814 RMB, 2.47 times the GDP per capita), which was approximately 1.78 times the median (99,906 RMB) in this report. Based on the above description, we think less than 3 GDP per capita for a QALY is an acceptable threshold in China; however, thresholds for different scenarios may need to be differentiated.

The median 99,906 RMB (14,479USD, 1.39 times the GDP per capita) of the overall sample in our study was smaller than the National Institute for Health and Care Excellence (NICE) threshold for end-of-life interventions of £50,000 (64,103USD, 1.56 times the GDP per capita in 2020) [27], and the ratio of the GDP per capita was close. In comparison to the WTP/QALY for patients with malignant tumors in other Asian countries, the median 99,906 RMB (14,479USD, 1.39 times the GDP per capita) of the overall sample in this paper was between the 11,031USD among patients with lung cancer in Vietnam in 2018 [28] and the 19,200–32,000USD among patients with solid tumors and the general population in the Kingdom of Saudi Arabia [29]. However, the ratio of the median WTP/QALY value to China’s GDP per capita was lower than the ratio of that in Vietnam (4.4 times the GDP per capita in 2017) [28] and close to that in Saudi Arabia (1–1.5 times the GDP per capita) [29]. In comparison to other studies that were based on the hypothetical scenario of incurable and maintenance therapy “restore and maintain in full health but need continuous administration”. The ratio of the GDP per capita in our study was larger than the €2,629 (0.17 times the GDP per capita in 2015) among Greek outpatients [30], €10,119 (0.44 times the GDP per capita, which was €22,758 in 2011) among Spanish patients in a health center [31, 32], SEK 120,000 andSEK 160,000 (0.56 and 0.74 times the GDP per capita, which was SEK 216,017 in 1995) among Swedish patients for hormone replacement therapy [33, 34]. Regarding studies among patients with chronic diseases with a hypothetical scenario of curable treatments (diseases including cervical spondylotic myelopathy and cerebral aneurysms [35], diabetes [36] and cardiovascular disease [37]), the ratio of the GDP per capita in our study was larger than that from King’s study in the American population (the WTP/QALY value was 12,500–32,200USD, 0.32–0.82 times the GDP per capita, which was 39,490USD in 2003) [35, 38] and larger than that in Moradi’s study in the Iranian population (1,191–5,043USD, 0.23–0.95 times the GDP for patients with diabetes; less than 1 times the GDP for patients with cardiovascular disease) [36, 37]. It seems that the WTP/QALY for malignant tumors may have a higher ratio to GDP per capita, and the ratio of GDP per capita for the curable scenario may be different between malignant tumors and other non-life-threatening chronic diseases.

Compared with the results of the 10 year installment payment plan scenario, the WTP/QALY for the lump-sum payment plan was significantly smaller (P < 0.05), indicating that the willingness to pay increased for curative treatments when budget constraints were loosened. In the current Chinese context, payment reforms improved the efficiency of health resource utilization focused on the supply side, such as the Diagnosis-Intervention Packet/Diagnosis Related Groups (DIP/DRGs) pilot program and execution in place, while significantly increased WTP/QALY values implied a solution from the demand side.

Regression analysis revealed that characteristics such as patient EQ-5D-5L health utility, annual household income per capita, and co-occurring chronic diseases had a significant impact on the WTP/QALY among patients and family members. In contrast to the results of study which was conducted in the Japanese general population [36, 39], the higher the utility value, the better the health status and the more people were willing to pay for a QALY, which was consistent with the results of studies from Vietnam on the lung cancer population [28] and from Iran among patients with heart disease [37]. Furthermore, in the regression analysis of patients, WTP/QALY values for patients with regular physical examinations were significantly higher, which was consistent with the results of the study conducted by Li Jieying et al. [40] on the willingness to pay for critical illness insurance; compared to patients with full-time employment, WTP/QALY values for patients who had retired were significantly higher, the reason might be a stable pension income and less financial burden. In the regression analysis of family members, age was a positively significant factor for WTP/QALY, implying the older patient's family was able to pay more; and compared to patients with full-time employment, WTP/QALY values were significantly higher among self-employees and students. This might be due to the fact that the impact on household income was limited since self-employed could work flexibly and students usually did not have income (receive the treatment at the expense of their family). Moreover, age was positively correlated with the WTP/QALY value in the regression analysis for the family member group, but there was no statistically significant difference for the patient group. This might be due to the financial burden of the treatment, the emotional toll of the disease, Chinese social custom that ‘‘the life span is destined and let nature take its course’’, that "people should be more willing to sacrifice for family members’’ and other social factors.

However, this study has some limitations. Firstly, the health utility value for chronic patients after the hypothetical curative treatment maybe biased. In our study, we cited the result from a study on older adults in Suzhou city (the EQ-5D-3L instrument was employed) due to the lack of a Chinese EQ-5D-5L chronic disease-based study. And the health utility of hypertension group was adapted (for chronic conditions not covered in Suzhou study), which had the highest health utility among the chronic conditions that were included. This would result in a lower WTP/QALY. Secondly, the sample’s representative in our study had certain limitations. The proportion of patients with liver cancer (1.8%) was much lower in our sample compared to the distribution ratio (9%) of malignant tumour types in China [2], which might be because patients with liver cancer tended to select cancer hospitals; The percentage of female patients in our study was greater than that of male patients, and the mean age (56.2 years) of the patients in our study was slightly lower than that of cancer patients in China (61.3 years) [24], these might be because the age of the patients in our study ranged from 18 to 75 years old (the gender ratio in our study is similar to that of cancer patients aged 15–55 in China (more females than males); and the sample does not include patients aged 75 or above, so the average age of the sample is smaller than that of the Chinese cancer population) [14]. In addition, we found that there was no statistically significant difference in WTP/QALY ratios among patients (family members) with different tumor types (Additional file 2). Because not all major tumor types were covered in our study, further studies with large sample sizes to cover major tumors and the characteristics of Chinese people with malignant tumors are needed.

## Conclusion

As far as we know, this is the first study to provide empirical evidence of the monetary value of a QALY among patients with malignant tumor in China. In our study, we estimated the WTP/QALY of the lump-sum payment and 10 year installment payment on the demand side by conducting a survey among Chinese individuals with malignancies in three cities with different GDP levels, and we found the WTP/QALY of 10-year installment payment for the curative therapy was larger. Furthermore, we compared the results with other studies on different diseases and hypothetical treatment scenarios, and the findings revealed that: the ratio of the WTP/QALY to GDP per capita may provide the thresholds by diseases (malignant tumors or other non-life-threatening chronic diseases) and hypothetical treatment scenarios, and a higher ratio of GDP per capita for malignant tumor therapies should be considered. Instead of utilizing a unified threshold or a general threshold range (such as 1–3 times the GDP per capita), decision making should also take into account differences originating from diseases and treatments.

## Supplementary Information

**Additional file 1: **A sample for CV task.

**Additional file 2: **Statistical analysis of WTP/QALY of different tumor types by subgroups.

## Data Availability

The datasets used and/or analyzed in the current study are available from the corresponding author upon reasonable request.

## Footnotes

1 Exchange rate for 2019: 1 USD = 6.91 RMB. Retrieved Apr 9, 2023, from https://data.worldbank.org.cn/indicator/PA.NUS.FCRF?locations=CN.

2 Exchange rate for 2020: 1 USD = 6.9 RMB. Retrieved Apr 9, 2023, from https://data.worldbank.org.cn/indicator/PA.NUS.FCRF?locations=CN.
