# Supplementary material for: Estimation of the value of curative therapies in oncology: a willingness-to-pay study in China
Source: Cost Eff Resour Alloc. 2023 Jun 5;21:37. doi: 10.1186/s12962-023-00442-y (PMC10243056; doi:10.1186/s12962-023-00442-y)
Supplement: Supplementary file 1 — Additional file 1: A sample for CV task. [file 12962_2023_442_MOESM1_ESM.doc]

Additional file 1. A sample for CV task

The average life expectancy of Chinese people is 80 years. Assuming the disease you ( or your family) are currently hospitalized for is a chronic condition, the disease can be fully cured by taking a new, painless, nontoxic and side effect free therapy without additional treatments (such as surgery, chemotherapy or radiation, etc). If you do not accept this novel therapy, you current state of health will be maintained. Are you willing to pay for this novel therapy at your own expense?

If the answer is Yes.

Please choose a price range within which you are willing to spend the most for this novel therapy. (Given your financial situation)

What is the most you are willing to spend within the price range you have chosen? (Please confirm the amount provided)

| 0-300,000 RMB |
| --- |
| 300,000-600,000 RMB |
| 600,000-900,000 RMB |
| 900,000-1,200,000 RMB |
| 1,200,000-1,500,000 RMB |
| 1,500,000-1,800,000 RMB |
| 1,800,000 RMB and above |

Your treatment cost mainly come from：

| Deposit | Income | Borrowing money | Asset sales | Others |
| --- | --- | --- | --- | --- |

If the answer is No.

The following are some probable reasons why you are unwilling to pay for this novel therapy:

| I can't afford it | The full cost should be paid by the government | Others |
| --- | --- | --- |

1. Are you willing to pay in installments if the cost of the novel therapy exceeds your financial capacity? (Patients under the age of 70 who respond positively to Question 1)

If the answer is Yes.

If you are paying for the therapy in ten-year installments, could you kindly specify a range within which you are willing to pay the maximum amount per year?

What is the most you're willing to spend each year within the range you've chosen? (Please confirm the amount provided)

| 0-30,000 RMB |
| --- |
| 30,000-60,000 RMB |
| 60,000-90,000 RMB |
| 90,000-120,000 RMB |
| 120,000-150,000 RMB |
| 150,000-180,000 RMB |
| 180,000 RMB and above |

If the answer is No.

The following are some probable reasons why you are unwilling to pay in ten-year installments:

| I can't afford it | The full cost should be paid by the government | Others |
| --- | --- | --- |
