# Supplementary material for: Estimation of the value of curative therapies in oncology: a willingness-to-pay study in China
Source: Cost Eff Resour Alloc. 2023 Jun 5;21:37. doi: 10.1186/s12962-023-00442-y (PMC10243056; doi:10.1186/s12962-023-00442-y)
Supplement: Supplementary file 2 — Additional file 2: Statistical analysis of WTP/QALY of different tumor types by subgroups. [file 12962_2023_442_MOESM2_ESM.docx]

Supplement 2. Statistical analysis of WTP/QALY of different tumor types by subgroups

| Respondent | Disease | Sample size | WTP/QALY_（r=5%）_ | | H | P |
| --- | --- | --- | --- | --- | --- | --- |
|  |  |  | Mean ± SD | Median |  |  |
| Patients group | Colorectal cancer | 102 | 288,130±965,704 | 75,449 | 9.000 | 0.174 |
|  | Lung cancer | 68 | 753,313±3,804,413 | 74,830 |  |  |
|  | Breast cancer | 105 | 175,796±286,172 | 72,770 |  |  |
|  | Gastric carcinoma | 47 | 382,843±968,765 | 89,857 |  |  |
|  | Cervical cancer | 65 | 122,453±179,027 | 54,890 |  |  |
|  | Nasopharyngeal carcinoma | 38 | 150,878±180,410 | 97,076 |  |  |
|  | Lymphoma | 37 | 327,034±612,975 | 136,868 |  |  |
| Family members group | Colorectal cancer | 58 | 406,597±762,367 | 150,117 | 3.173 | 0.366 |
|  | Lung cancer | 74 | 344,361±523,419 | 154,898 |  |  |
|  | Breast cancer | 37 | 333,433±704,253 | 99,906 |  |  |
|  | Gastric carcinoma | 45 | 293,967±649,795 | 63,467 |  |  |
